# Contested role boundaries and professional title: Implications of the independent review of podiatric surgery in Australia

**DOI:** 10.1002/jfa2.70007

**Published:** 2024-10-18

**Authors:** Alan M. Borthwick, Susan Nancarrow, Ivan Bristow, Catherine Bowen

**Affiliations:** ^1^ School of Health Sciences Faculty of Environmental and Life Sciences University of Southampton Southampton UK; ^2^ School of Clinical Sciences Queensland University of Technology Brisbane Queensland Australia; ^3^ Allied Health Workforce HealthWork International Brisbane Queensland Australia; ^4^ School of Health and Human Sciences Southern Cross University East Lismore New South Wales Australia; ^5^ Podiatry Department Christchurch Foot Clinic Christchurch UK; ^6^ Podiatry Department Health Sciences University Bournemouth UK; ^7^ Podiatry Department School of Health Sciences Faculty of Environmental and Life Sciences University of Southampton Southampton UK

**Keywords:** gatekeeper, interprofessional conflict, jurisdictional disputes, medical dominance, podiatric surgeon, podiatric surgery, social closure, symbolic capital, symbolic devaluation, symbolic violence

## Abstract

**Introduction:**

In October 2023, the Podiatry Board of Australia commissioned an independent review of the regulation of podiatric surgery in Australia, with a remit to re‐evaluate the regulatory framework, identify any risks to patient safety and recommend improvements to public protection. It reported in March 2024 and set out 14 key recommendations. The review was prompted by a number of complaints about podiatric surgeons but also reflected calls for reform by the medical profession and several critical media reports. This paper sets out to examine the review report, alongside the concerns of the medical profession and the media articles expressed within it, through the lens of an established sociological framework focused on interprofessional conflict and the contested use of professional titles.

**Methods:**

As a review rather than the research paper, the Independent Review of Podiatric Surgery (the ‘Paterson Report’) served as data for the sociological analysis, adopting a Neo‐Weberian and Bordieuan framework to examine the strategies adopted by the medical profession and media reports cited in the report, consistent with the exercise of professional power.

**Results:**

The sociological analysis provides insights into the ways in which professions seek to maintain symbolic, social, cultural and economic privileges and rewards through the exclusion of competitors, using strategies such as social closure, symbolic violence, symbolic devaluation, gatekeeper roles, and jurisdictional disputes.

**Conclusions:**

The review report acknowledges the influence of the medical profession and its opposition to the practice of podiatric surgery and use of the title ‘podiatric surgeon’. The arguments made and strategies deployed are consistent with those found in the wider literature. In light of these findings, the implications for the future of podiatric surgery are considered in terms of professional practice, use of professional title, and access to public funding.

## 1 BACKGROUND AND CONTEXT

In March 2024, an independent review of the regulation of podiatric surgery in Australia was published, having been commissioned by the Podiatry Board of Australia (PBA) and the Australian Health Practitioner Regulation Agency (AHPRA) [1]. It was ‘triggered by the high rate of complaints or notifications about podiatric surgeons’ and followed in the wake of ‘media articles and calls for reform—mainly from orthopaedic surgeons’, drawing into question the registration of podiatric surgeons under the National Registration and Accreditation Scheme [1]. Interprofessional competition within healthcare is widely acknowledged [2, 3, 4, 5, 6, 7, 8, 9], and tensions specifically between orthopaedic and podiatric surgeons have been recognised over many years [10, 11, 12, 13, 14, 15, 16]. Examining the recent review (the ‘Paterson Report’) through the lens of an explanatory sociological framework focused on interprofessional tension sheds light on the strategies adopted in areas of disputes [2, 3, 4, 5, 6, 7, 8, 9]. ‘Jurisdictional disputes’ [2] tend to focus on contested task domains and role boundaries as well as the use of titles, which are defended by deploying ‘social closure’, a means to ensure the exclusion of competitors from access to these privileges [3]. This is achieved through defining recognised expert credentials and titles, obtaining legislative or regulatory controls, and controlling the profession's narrative with government, state authorities and public [3]. Ultimately, success depends upon the ability of a profession to harness the support of these ‘powerful elites’, which are sufficiently influential to ensure the profession's narrative is accepted [17]. Medicine exerts considerable social and cultural authority (as the hegemonic authority in healthcare) that has been effective in protecting its pre‐eminence, often referred to as medical dominance [3, 4, 5]. Interprofessional competition has been characterised as ‘regulated peaceful conflict’, reflecting the way in which professions act to defend their role boundaries or symbolic capital (titles), in clear contrast to contemporary demands for workforce flexibility in the face of staff shortages and increasing demand from an ageing population [18, 19].

These two drivers collide—the professional desire for exclusive monopolistic control over key professional boundaries and titles, ranged against the need for health services to adapt to meet demand [20, 21, 22]. One seeks the maintenance of long‐established hierarchical norms, and the other demands new and innovative solutions in which the medical profession does ‘not have a monopoly on care’ [23].

## 2 MAIN TEXT

The Paterson review aimed to clarify and examine the basis of the concerns expressed, through an information gathering exercise and public consultation, including the views of patients and the wider public, regulatory authorities, the medical profession, the podiatry profession, insurance companies and relevant education providers [1]. Deploying Neo‐Weberian and Bordieuan sociological theory reveals the complex mix of motives and strategic responses that characterise interprofessional disputes, and the arguments presented in the report mirror many of the same concerns found in other cases, such as in the UK [11, 12, 14, 16].

Indeed, the review demonstrates that, whilst jurisdictional disputes continue to be a ‘fundamental fact of professional life’ [2], the practice of podiatric surgery is firmly established as a viable, safe and effective option. Attempts by the medical profession to exclude, control or limit podiatric surgery in Australia have largely failed, mirroring the UK, where contemporary opposition is now more focused on the use of professional title [11, 14]. Whilst at face value, the review may appear to challenge some aspects of podiatric surgical practice, on closer inspection it clearly offers support for its continued growth, as well as tacit support for future public funding as part of an integrated public service. However, significant challenges lie ahead, given the ‘gatekeeper’ role of the medical profession.

### 2.1 The Paterson Report: ‘Gatekeeping’ and the future of podiatric surgery

Full and effective utilisation of health practitioners requires access to appropriate training, regulation and funding. The Patterson report demonstrates that podiatric surgeons have successfully negotiated the first two of these requirements [1]. They have established an accredited and recognised training programme and achieved professional closure through regulation with AHPRA. However, they are still restricted from working to their full capacity due to a lack of access to government (Medicare) funding for their services and restrictions on access to public hospitals.

This is where the concept of the gatekeeper becomes crucial [24]. While the scope of practice of podiatric surgeons has effectively met the training and regulatory requirements necessary to practice, the medical profession still holds significant power to restrict the work practices of other professions. They act as gatekeepers, controlling access to essential tools and resources, including government funding and hospital privileges.

To understand these constraints, it is crucial to consider Bourdieu's concept of the field [24]. This framework emphasises the dynamic relationships that give rise to social action within a given social space. According to Bourdieu, any social action can be understood by identifying the relations and structures of domination in that particular field. All fields are sites of struggle, constituted by a set of relations between various positions that reproduce the field itself.

In healthcare, the key actors include individual medical doctors, government ministers, other health professionals and organisations such as specialist colleges, pharmaceutical companies, professional associations and insurers. These actors hold varying degrees of power and influence, often rooted in historical and structural advantages. The medical profession, in particular, has long held a dominant position, enabling it to control decision‐making processes that affect other health professions.

For example, the medical profession's influence over government policy can impact which services are eligible for Medicare funding. Without Medicare funding, podiatric surgeons find it challenging to offer their services to a broader population, limiting their practice to private patients who can afford out‐of‐pocket costs. Similarly, restrictions on access to public hospitals prevent podiatric surgeons from performing surgeries in these settings, further limiting their practice scope and the public's access to their specialised services.

These gatekeeping actions are not merely bureaucratic hurdles but are rooted in the power dynamics within the healthcare field. By controlling access to critical resources, the medical profession maintains its dominance, reinforcing a hierarchical structure that perpetuates inequality among health professions. Addressing these issues requires a re‐examination of the power relations within the healthcare field and implementing policies that ensure equitable access to training, regulation and funding for all health practitioners.

In conclusion, while podiatric surgeons have demonstrated their competence through rigorous training and regulatory compliance (As noted throughout the Paterson Report: ‘…there is no basis for a restriction of the scope of practice of podiatric surgeons’; ‘…concerns about the quality of education and training of podiatric surgeons are not supported by the evidence’; ‘…there is not sufficient evidence of non‐compliant advertising leading to harm to warrant an audit’ [1]), their full and effective utilisation is hindered by systemic gatekeeping. Understanding and addressing these barriers through the lens of Bourdieu's concept of the field can help promote a more equitable and efficient healthcare system.

Although careful to state that it is ‘beyond the scope’ of the review to make a formal recommendation on public funding for podiatric surgery, it is viewed as a goal worth pursuing, as the review recommends that the PBA and AHPRA ‘write to health ministers’, and, with their support ‘work with the Australian Government…to explore options to integrate…into the broader healthcare system’ [1].

### 2.2 The Paterson Report: Further points

Certain further points merit attention. The recommendation that mandatory endorsement for scheduled medicines certification (or ESM) should be introduced reflects the importance of non‐medical allied health prescribing to effective patient management, now widely supported in the literature [25, 26, 27, 28, 29, 30, 31, 32].

Importantly, medical power is also exercised through its ability to control the media narrative. Articles in the Sydney Morning Herald and The New Age, published between December and March 2024, cited by Paterson, report patient cases of surgical complications which are directly linked to concerns over podiatric surgical education and training, most notably the absence of ‘medical degrees’ held by podiatric surgeons [33, 34, 35, 36]. This argument reflects the way the media broadly accepts and reproduces the medical narrative in matters of healthcare, sometimes called ‘doctoring the media’ [37, 38]. Equating surgical complications in podiatric surgery with a lack of education and training in medicine is premised on the notion that a surgeon without a medical degree is unqualified [14]. Full training in podiatric surgery is not deemed sufficient or acceptable. For Bourdieu, this is a manifestation of symbolic devaluation, deployed by a more established profession to undermine a threat from a less powerful profession [36, 37, 38, 39, 40].

Whereas the review considers the accreditation standards for the education and training programmes to be ‘broadly consistent with’ those used by the Australian Medical Council, it identifies a need to strengthen the accreditation assessment teams to include a member with surgical training and experience (implying a medical professional). Whilst the review views this as an opportunity for more collaborative working relationships with the medical profession, it nonetheless acknowledges that ‘Vehement opposition from orthopaedics…and resistance from podiatric surgeons to the involvement of a competing speciality’ makes the prospect ‘unrealistic’. It cleverly envisages an alternative option, where individuals ‘from other medical specialties would be willing to help’. Indeed, the review broadly acknowledges the ‘outright hostility from orthopaedic surgeons’, and urges caution and a ‘need for proportionality in the regulatory responses’ to orthopaedic complaints about podiatric surgery. Thus, there is a tacit understanding of the underlying strategies at work when groups compete over role boundaries and task domains.

### 2.3 The issue of title: ‘Podiatric surgeon’

On the matter of professional title, the review recommends that the PBA seeks health ministers' approval to change the professional title from ‘podiatric surgeon’ to an alternative, such as ‘surgical podiatrist’. The specialist title of ‘podiatric surgeon’ is recognised in the Health Practitioner Regulation National Law (2009) but has consistently been opposed by the medical profession [1]. The title was the ‘single biggest issue raised by orthopaedic surgeons’ in their submissions to the review consultation [1]. The review concludes that patients may be confused by the title ‘podiatric surgeon’ and do ‘believe and expect’ that their podiatric surgeon will ‘have a degree in medicine’. However, the review is mindful of the fact that a similar argument was used in 2009 during the consultation phase leading up to the introduction of the National Scheme. The Australian Orthopaedic Association claimed that the title would mislead patients and compromise patient safety, but the Australian Workforce Ministerial Committee ‘rejected’ those assertions [1].

In the current context, the medical profession can, however, point to the changes made to the National Law in 2023, which *do* limit the use of the title ‘surgeon’ to those medical practitioners who are registered in one of the specialties of surgery, obstetrics and gynaecology, or ophthalmology (in a bid to better regulate cosmetic surgery) [41, 42]. However, this provision applies only to medically qualified doctors and not to podiatric or dental surgeons (which are omitted). Nevertheless, it allows the medical profession to argue that securing such a change enhances the likelihood that the public will expect anyone using the title ‘surgeon’ to be medically qualified [1].

Titles are important assets to professions (symbolic capital), reflecting power, prestige and status. Bourdieu's conceptual framework permits a crystal‐clear analysis of the dispute between two competing professions over the use of professional title [39, 40, 43, 44, 45]. Titles are symbolic capital and are defended through the exercise of ‘symbolic violence’ (Symbolic violence is the imposition of systems of symbolism and meaning [i.e., culture] upon groups in such a way that they are experienced as legitimate. The legitimacy obscures the power relations. [See Jenkins, R. Pierre Bourdieu. London: Routledge. 2002].) [39, 40, 43, 44, 45]. This allows the medical profession to reassert its cultural and symbolic authority over the domain of surgery. It acts to reinforce the taken‐for‐granted assumption that those practising surgery must obviously be medically qualified, thus concealing the underlying exercise of power (the ‘doxa’ in Bordieuan parlance) [39, 40, 44]. This enables the dominant profession to assert that the use of the title by competitors is misleading. One effective means of achieving this aim is to devalue the competing profession (symbolic devaluation), commonly manifest as pejorative criticisms, implying wilful deceit, incompetence or inadequate training [40]. This is illustrated in the alternative titles suggested by orthopaedic surgeons in their submissions to the review, such as ‘podiatric technician’, or in describing the notion that podiatric surgical training is safe as ‘ridiculous’ [46].

There is an important corollary to the claim that the title ‘podiatric surgeon’ is protected under the National Law. The Act explicitly lists its protected titles, including ‘podiatrist’ and ‘chiropodist’. However, ‘podiatric surgeon’ is not included in this list; instead, it is recognised via a slightly different mechanism within the Act (Part 2, Section 13, 2(b).). The Health Ministers Meeting (HMM; formerly the Ministerial Council) provides separate approval for specialist titles, usually granted on the recommendation of a National Board [47]. Such an approval may therefore, in theory, be revoked via the same mechanism (by a decision of the HMM), without altering the text of the National Law (Thanks are due to Mr Nick Studdert, Consultant Podiatric Surgeon and former lawyer, for his advice on this point.). This may allow effective lobbying to influence the final ministerial decision.

## 3 CONCLUSION

The Podiatry Board has accepted all the recommendations in the review [48, 49]. It must first undertake a full consultation before seeking Health Ministers' approval [48, 49]. This will enable each case to be made in advance, either to retain the title or remove it. Given the nature of symbolic violence, a challenge to the dominant discourse of medicine may seem an insurmountable challenge. Yet, as the review points out, earlier challenges have been successfully resisted. It also acknowledges the fact that podiatric surgeons have ‘legitimately’ used the title for the last 15 years or more [1]. Interestingly, the assertion that the title ‘surgical podiatrist’ would reduce consumer confusion remains debatable. There is no real evidence to support such a claim, nor that ‘podiatric technician’ or ‘operative podiatrist’ would bring greater clarity.

The review report sheds light on contemporary interprofessional conflicts, and the pursuit of exclusive privileges by one group at the expense of another. As in the UK, challenges to the scope of practice of podiatric surgery are no longer sustainable or successful [14]. Thus, the conflict is now focused on the struggle for symbolic legitimacy via professional title, which acts as a ‘distinctive mark’ that draws its value from its position within a hierarchically arranged system of titles [43]. As Bourdieu himself stated,

“… it is not the relative value of the work that determines the value of the name, but the institutionalised value of the title that can be used as a means of defending or maintaining the value of the work” [43].

## AUTHOR CONTRIBUTIONS

**Alan M. Borthwick**: Conceptualization; data curation; formal analysis; methodology; writing—original draft; writing—review and editing. **Susan Nancarrow**: Conceptualization; data curation; formal analysis; methodology; writing—original draft; writing—review and editing. **Ivan Bristow**: Writing—review and editing. **Catherine Bowen**: Writing—review and editing.

## CONFLICT OF INTEREST STATEMENT

The authors declare that they have no competing interests.

## ETHICS STATEMENT

Not applicable. No human participants were involved in this policy review paper.

## CONSENT FOR PUBLICATION

Not applicable (no individual data is included).

## Data Availability

Not applicable (as a review/policy paper).
